# Developing dimensions for a new preference-based quality of life instrument for older people receiving aged care services in the community

**DOI:** 10.1007/s11136-020-02649-5

**Published:** 2020-09-29

**Authors:** Jenny Cleland, Claire Hutchinson, Candice McBain, Ruth Walker, Rachel Milte, Jyoti Khadka, Julie Ratcliffe

**Affiliations:** 1 College of Nursing and Health Sciences, Flinders University, Adelaide, Australia; 2 John Walsh Centre for Rehabilitation Research, Faculty of Medicine and Health, The University of Sydney, Sydney, Australia; 3 Healthy Ageing Research Consortium, Registry of Older South Australians (ROSA), South Australian Health and Medical Research Institute (SAHMRI), Adelaide, Australia

**Keywords:** Quality of life, Older people, Aged care services, Preference-based instrument, Economic evaluation

## Abstract

**Purpose:**

To identify the salient quality of life characteristics relevant to older people in receipt of community aged care services in order to develop dimensions for a draft descriptive system for a new preference-based quality of life instrument.

**Methods:**

Forty-one in-depth semi-structured interviews were undertaken with older people (65 years and over) receiving community aged care services across three Australian states to explore quality of life characteristics of importance to them. The data were analysed using framework analysis to extract broader themes which were organised into a conceptual framework. The data were then summarised into a thematic chart to develop a framework matrix which was used to interpret and synthesise the data. Care was taken throughout to retain the language that older people had adopted during the interviews to ensure that appropriate language was used when identifying and developing the quality of life dimensions.

**Results:**

The analysis resulted in the identification of five salient quality of life dimensions: independence, social connections, emotional well-being, mobility, and activities.

**Conclusion:**

This research finds that quality of life for older people accessing aged care services goes beyond health-related quality of life and incorporates broader aspects that transcend health. The findings represent the first stage in a multiphase project working in partnership with older people to develop a new preference-based instrument of quality of life for informing quality assessment and economic evaluation in community aged care. In future work, draft items will be developed from these dimensions and tested in face validity interviews before progressing to further psychometric testing.

## Introduction

The policy and practice landscape for the Australian aged care sector, in common with the aged care sectors of many other countries, is changing with a significant increase in recent years in the incidence of older people accessing community aged care services [1]. In Australia, the home care packages (HCP) programme is the fastest growing sector of the aged care system with 116,800 people receiving a HCP in 2017–2018 compared to 97,200 in 2016–2017 [2]. This sector is predicted to increase markedly in the coming decades due to increasing numbers of people living at home with frailty, cognitive decline and dementia [3]. In Australia, the aged care sector is currently the subject of a Royal Commission that is placing an international spotlight on the shortfalls of the system and which is likely to highlight the need for significant policy reform to drive improvements in quality and efficiency when it issues its final recommendations at the end of this year [4]. A suite of recent systematic reviews have identified the paucity of economic evaluation evidence internationally, [5–8] yet economic evaluation forms a much needed component for policy reform to drive quality improvements and ensure that resources allocated across the aged care sector are targeted to services and programmes which maximise the quality of life (QoL) of older people [6, 7, 9].

Cost-utility analysis is a widely applied economic evaluation framework which synthesises costs and outcomes into a cost-utility ratio, whereby outcomes are most often assessed through the calculation of quality-adjusted life-years (QALYs). QALYs measure and value QoL on the zero (equivalent of being dead) to one (full health) QALY scale and are typically generated through the application of preference-based QoL measures. A recent review of the development and application of generic preference-based instruments with the older population across health and social care sectors by Cleland et al. [10] highlighted the EQ-5D-3L [11] and EQ-5D-5L [12] as the most widely used measures applied with the older population to date. These measures that focus on health-related QoL were applied in 137 studies. The Adult Social Care Outcomes Toolkit (ASCOT), [13] a preference-based measure of social care related QoL and the Investigating Choice Experiments Capability Measure for older people (ICECAP-O), [14] a measure of capability for older people, have also been applied in a number of studies (6 and 13, respectively). Research conducted by Van Leeuwen et al. [15] exploring the content validity and feasibility of the EQ-5D-3L, ASCOT and ICECAP-O with older adults found that some of the dimensions in these measures were not deemed to be relevant and none of the measures captured all the dimensions that older people thought were important to their quality of life. The most important aspect of an instrument is its content relevance. None of the existing preference-based measures are specific to older people accessing aged care. Previous work by Ratcliffe and colleagues [16] published in this journal found that the preferences of younger and older people in relation to the relative importance of dimensions of QoL are not the same. Whilst this research found some importance differences in preferences between younger and older adults the study did not collect any qualitative data to further explore and examine why these differences existed. A need for further research to be undertaken to explore the concept further and to develop a preference-based measure for older people accessing aged care were the main recommendations from this previous study. Ratcliffe’s work and that of others has highlighted that older people’s views and preferences about what encapsulates QoL from their perspective goes beyond health status incorporating wider dimensions such as independence, control, social relationships, and dignity [17, 18]. If economic evaluations conducted in aged care are to accurately assess the value of competing interventions, it is important that the defining characteristics of QoL for older people are adequately captured within QALY calculations. Our research aims to address this gap by developing an older person-specific preference-based QoL measure for application in quality assessment and economic evaluation in aged care.

Most of the existing preference-based QoL and patient-reported outcome (PRO) measures more generally have been developed using a professional-centred approach based upon the literature and/or the views of experts [19]. However, the involvement of the population of interest using a person-centred approach from inception has recently been encouraged in developing PRO measures [20]. This approach has also recently been incorporated in the development of a new preference-based QoL measure for paediatric populations [21] and was utilised in the development of the ICECAP suite of measures for assessing capabilities in adult and older populations [17, 22]. Whilst the ICECAP-O was developed for application with older people using a similar approach to the one adopted here, it is a measure of capability developed with older people living in the community in the UK and has Sen’s capability approach as its fundamental theoretical foundation [23, 24]. In contrast, this study sought to partner more specifically with aged care organisations and older people in Australia receiving community aged care services to develop a new preference-based measure of QoL. A person-centred approach was applied to directly facilitate the use of appropriate language and content relevant to older people receiving community aged care services, thereby increasing its content validity and relevance [25–27]. This paper reports on the first stage of our multiphase research project to develop an older person-specific preference-based QoL measure for application in aged care. This phase involved identifying the salient QoL dimensions relevant to older people in receipt of community aged care services to develop the draft descriptive system for the measure.

## Methods

### Recruitment

Participants were recruited from five aged care organisations providing community aged care services to older people in metropolitan and rural areas across three Australian states (South Australia, Victoria, and New South Wales). Eligibility to participate in the research was based on the following criteria: aged 65 years and over; in receipt of a government HCP; ability to communicate in English; normal cognitive functioning through to mild cognitive impairment/mild dementia (assessed by the aged care organisation using the Psychogeriatric Assessment Scale Cognitive Impairment Scale (PAS-Cog [28])); and ability to provide informed consent. Purposive sampling was utilised to ensure a representative sample of older people receiving HCPs participated in the study. The research team provided the eligibility criteria and an overview of the demographics of participants in Australia receiving HCPs to the aged care organisations who recruited using this criterion enabling our sample to be broadly representative.

The aged care organisations approached potential participants in the first instance and provided them with a letter detailing the research and a participant information sheet. After one week, the organisations re-contacted the participant to ask if they were interested in taking part and to gain verbal consent for the researcher to approach the participant. If consent was gained, the organisation gave the contact details of the older person to the research team. The researcher then contacted the participant via telephone to confirm their willingness to participate, answer any questions, and to arrange a face-to-face interview.

In total, 41 qualitative interviews were undertaken with participants who were receiving community aged care services in South Australia, New South Wales, and Victoria. The participants were interviewed in their choice of setting with all the participants choosing to be interviewed in their own home. The interviews were conducted by two researchers with prior experience of interviewing older people in this context. On arrival, the researcher discussed the participant information sheet, explained the interview process, answered any questions, and gained written consent. The interview duration ranged from 21 to 69 min (mean = 33 min).

### Interviews

The in-depth semi-structured interviews consisted of three stages. The researchers followed an interview schedule to ensure the interviews were conducted in the same manner. At the first stage the researcher began the interview by asking the participant ‘What does QoL mean to you?’ to initiate a discussion. This question was then followed by open-ended questions about QoL in general to explore the meanings around the term and characteristics that were important to the older person. These open-ended questions enabled the participant to initiate discussion about the issues relating to QoL which were most pertinent to them, and for the interviewer to then probe further to understand the factors and issues relating to QoL. The participant was then asked questions about the aged care services they were receiving to understand how their support impacted upon their QoL. The second part of the interview consisted of the presentation of a series of 12 cards reflecting different dimensions of QoL. The card labels and descriptors are based on the content of the descriptive systems of the EQ-5D, the Assessment of Quality of Life (AQoL) and the ASCOT instruments and were developed and employed in the previous study by Ratcliffe and colleagues to ascertain older and young person preferences for attributes of QoL [16]. Each participant was presented with the 12 cards, each card displaying a single QoL dimension (independence, safety, social relationships, hearing, vision, mental health, sleep, physical mobility, self-care, dignity, control, pain) with a brief description to explain its meaning. The cards were used to probe the participant about the different dimensions of QoL that they deemed important to promote further discussion on the impact of the dimensions on their quality of life. Participants were also provided with the opportunity to discuss any other aspects of QoL that may not have already been discussed in the interview. The final stage involved the participant completing the EQ-5D-5L (self-complete version) and a short questionnaire that included questions about the participant’s socio-demographics and their care. The EQ-5D-5L and the socio-demographic questionnaire were printed in large font size to accommodate participants with visual impairment.

### Analysis

The analysis was guided by the purpose of the research which was to understand the QoL characteristics important to older people in order to identify salient candidate dimensions for the development of a descriptive system for a new preference-based QoL measure for older people in receipt of aged care. The dimensions were developed from stage one of the interviews with stage two providing further data to illustrate the impact of the dimensions on participant’s quality of life. Data saturation was assessed by the two researchers through conducting their own interviews and reading each other’s interview transcripts as the data collection progressed to establish when no new themes were emerging. Saturation was reached prior to the 41 interviews being conducted but it was decided that all the interviews would still be carried out to increase confidence in the data.

Interviews were transcribed verbatim by a Flinders University approved transcriber and the data were analysed using framework analysis [29–31]. Framework analysis as a technique was initially developed in the 1980′s in a social policy context for large-scale policy research as a response to the growth of qualitative research being undertaken [31]. However, it has now been used extensively in analysing qualitative data in health services research, [32, 33] including in QoL research [34–38], in the development of the CHU9D, a preference-based HrQoL measure for children [21, 39] and in the ICECAP-O, a preference-based measure of capability for older people [23]. The software package NVivo version 12 [40] was used to manage the qualitative data and analysis.

Framework analysis is a structured and vigorous approach to analysing qualitative data and consists of several stages. The first stage of familiarisation involved reading and re-reading the interview transcripts to become familiar with the data. The two authors who conducted the interviews (JC and CMB) read all transcripts whilst the other authors read a subsample of transcripts each. In the second stage, two independent coders (JC and CMB) analysed all the transcripts and developed an initial set of codes. At stage three, all members of the authorship team attended a workshop where the initial codes and quotes were examined, discussed, and collated into broader themes to form an agreed analytical framework.

In stage four, known as *indexing,* the two initial coders applied the analytic framework, collating participants’ quotes under the agreed themes. Stage five involves a process known as *charting* and is a unique aspect of framework analysis. In this stage, the coders develop a framework matrix which consists of rows (participants) and themes (columns) with each cell containing data summaries, thereby reducing the data whilst retaining the original perspectives of participants. In this way, the research team could examine the data at different levels of abstraction to aid synthesis and interpretation at a second workshop of the authorship team where the charts were reviewed. The framework approach therefore assisted in maintaining an analytical trail. Throughout the analysis, care was taken to retain the language that older people had adopted during the interview to ensure that appropriate language was used when developing the dimensions and which can subsequently be drawn upon to develop the draft items for the QoL measure.

### Statistical analysis

Summary statistics for the sample were generated as simple frequencies and percentages using SPSS, version 25.0 [41] and are presented in Table 1. Health state utility values for the EQ-5D-5L were generated from a pilot scoring algorithm based on a DCE approach in an Australian general population sample [42]. A final Australian general population scoring algorithm for the EQ-5D-5L is currently in development but is not yet publicly available. Utility scores range from − 0.676 to 1 where health states with a score less than 0 are considered worse than death.

**Table 1:** Participant characteristics (*n* = 41) and Australian home care population [1, 43]

Study participants	*N* (%)	Australian home care population	%
Sex		Sex	
Male, *n* (%)	11 (27)	Male, %	36
Female, *n* (%*)*	30 (73)	Female, %	64
Age		Age	
65–79, %	12 (29)	65–79, %	35
80–89, %	21 (51)	80–89, %	47
> 90, %	8 (20)	> 90, %	18
Living arrangements			
Living Alone, *n* (%)	26 (63)		
Living with spouse/partner, *n* (%)	12 (29)		
Living with other relatives, *n* (%)	2 (5)		
Living with others (not relatives), *n* (%)	1 (2)		
Home care package level		Home care package level	
Level 1 (basic care needs), *n* (%)	2 (5)	Level 1 (basic care needs), %	9
Level 2 (low care needs), *n *(%)	19 (46)	Level 2 (low care needs), %	44
Level 3 (intermediate care needs), *n* (%)	8 (20)	Level 3 (intermediate care needs), %	19
Level 4 (high care needs), *n* (%)	12 (29)	Level 4 (high care needs), %	28
Self-reported health			
Excellent, *n* (%)	2 (5)		
Very Good, *n* (%)	4 (10)		
Good, *n* (%)	20 (49)		
Fair, *n* (%)	12 (29)		
EQ-5D-5L Score, mean (SD) EQ-VAS Score, mean (SD)	0.56 (0.28) 66.88 (18.46)		

## Results

### Participant characteristics

Forty-one interviews were completed with older adults aged 68 to 95 years old. Four participants chose to have a family member present during the interview. These individuals did not formally participate in the research or influence the participant’s response in any way. Approximately three-quarters of the sample were female (73%). Most of the older adults lived alone (63%) with just under a third (29%) living with their spouse or partner. Five percent were receiving a level 1 HCP, nearly half (46%) a level 2 HCP, a fifth (20%) a level 3 HCP, and just under a third (29%) a level 4 HCP. The socio-demographic characteristics (age and sex data only available for comparison) and the distribution of HCP levels across our study sample are broadly representative of the population of older people currently receiving HCPs in Australia [1, 43]. Participant’s health-related QoL as approximated by the EQ-5D-5L using the Australian general population-specific scoring algorithm was on average significantly lower (mean 0.56, SD 0.28) than for the general population of similar age range (mean 0.85, SD 0.16) [44]. This finding was not unexpected given that the sample comprised dependent older people in the community receiving aged care services (Table 1).

### Dimensions

The analysis produced five salient dimensions that were consistently identified: independence, social connections, emotional well-being, mobility, and activities. Each of these dimensions will be discussed in more detail below. Relevant quotes illustrating the themes are presented in Table 2.

**Table 2:** QoL Dimensions illustrated by quotes

Dimension	Quote
Independence	*Quality of life? Well, that means to me that I still have independence. I like to be able to do things for myself* (Female, 91 years) *The worst thing about getting old, is your lack of independence.* (Female, 87 years) *Well, I like to be independent. I like to be able to look after myself as much as possible, but it is nice to have the help when you feel that you can’t* (Female, 83 years) *Well, I think quality of life to me means everything. It means independence, to be in charge of yourself. Yeah, that probably just sums it up in a couple of words, you know, to have that total independence really… just be in control; that’s really, really important to me. Not to have other people make decisions. I make the decisions where it relates to me* (Female, 76 years) *Well, I like having control over what I do. I don’t want to have people telling me what to do* (Male, 78 years)
Social connections	*You need a few good friends and you need good family, supportive family, and also neighbours are important when you’re so much at home. I’m very fortunate, I have nice neighbours and I have friends and family who are very, very supportive* (Female, 76 years) *One girl comes in and does the cleaning, two hours, and we have a little bit of a chatter and cup of tea. The other girl comes in and we go shopping and have a cup of coffee out so that’s my enjoyment as far as the week’s concerned. It breaks up the week.* (Female, 95 years) *I don’t have social relationships with people except for the people who come here, carers* (Male, 82 years) *I’ve just lost a very dear friend. Seventy-five years we’ve been friends. That was only two weeks ago. It’s hard to watch them go, you know* (Female, 85 years) *I think [Names], they are simply fantastic friends. I tell them what I do. I tell them my life history; they tell me their life history and I’ve never had such good friends…Normally I would see them on a daily basis* (Male, 78 years)
Emotional well-being	*When I first lost my sight I went into depression and being a bright person that was disastrous but it took me two years to accept that I couldn’t – I was a dressmaker, I was a china painter, I did all sorts of fine crafts, taught fine crafts and then not to be able to do any of it, that was very – it took a lot out of me.* (Female, 93 years) *I do suffer with the anxieties and I do get worried. Last year I became very depressed, just through other family members that tried to make our lives very difficult* (Female, 76 years) *I do sometimes feel, not depressed but I feel a bit anxious about things* (Female, 84 years) *I have the horrors about going into care… I don’t know how I’d cope with that* (Female, 91 years) *I’m happy and happy is quality of life* (Male, 78 years)
Mobility	*One quality of life that I miss is my mobility. That’s being unable to walk very far. It’s lack of ability that’s the greatest lack that I have. I am constrained from doing what I want to do, you know, because physically I’m constrained, not mentally but physically.* (Female, 87 years) *That’s [physical mobility] a little bit difficult at the moment. Apart from the ankle I could get around all right but it’s just a bit awkward now. As I say, I used to walk around the village every day. I can’t walk properly now without pain* (Male, 84 years) *I can’t get around very much on my feet. I have a stick and my walker so that’s very important to me* (Male. 81 years) *Physically when you go to do it you no longer can do it. Now, up until a couple of years ago I could walk around and do all different things, just took it for granted* (Female, 76 years)
Activities	*Well, this probably might attribute to some people but I go to what’s called [name]here and we don’t learn craft because that’s behind us now, we all know knitting, crocheting, cross stitch, all that sort of thing, and that is my happiest day of the week, Wednesday morning. We have so much fun, we just talk to one another and laugh* (Female, 84 years) *I’m a person that likes to be doing something…I like to be active. I like to – I paint as a hobby… I like to have interest in things. I’m a member of the jazz club. I don’t always get there as often as I’d like to…I just like to keep busy* (Female, 85 years) *I do lots of crosswords and I watch quiz shows. I’ve got to keep my mind busy. I think that’s a really important thing when you’re getting older, to keep your brain moving, you know, keep it going.* (Female, 80 years) *I think there’s things I want to do now that I haven’t done for many, many years. I want to get back to singing. My voice has gone, and I want to get it back so I’m going to be getting back on the keyboard to keep my voice going. That’s important to me because I was a singer, you see* (Female, 80 years) *I like to get out and – with my volunteering job at the library and relationships [at the] keep fit class and all those sorts of things* (Male, 81 years)

### Independence

The importance of being independent was discussed by all participants as a key attribute of their QoL. Most participants spoke about their desire to retain their own independence during the ageing process by continuing to do as much as possible for themselves including making their own decisions. This was of particular importance to participants as they wanted to retain control over their own lives and minimise their reliance on other people. In addition, some participants noted how much they valued the support they received from aged care services to help them to remain living at home independently. Whilst some participants receiving high-level HCPs acknowledged they would not be able to remain at home without the support they currently received, others receiving lower level HCPs were also grateful for help with various daily tasks and activities of daily living that they recognised that they had difficulties undertaking and/or were no longer able to do.

### Social connections

Almost all participants spoke about being connected to family and friends and how good relationships with those close to them were important for them to experience a good quality of life. Family was especially important to many participants and they looked forward to contact with family and valued their support. Relationships with friends were also important for many participants. Contact with friends was experienced face to face and via the telephone. Several participants also discussed being disconnected from family and friends due to health restrictions, death of loved ones, and family disagreements. These social disconnections often caused participants to worry and become anxious, and in some cases were a cause of great upset. For some participants, contact with their paid carers was the only social connection they experienced, and they looked forward to their visits. Some participants expressed that they had developed close bonds with these carers and considered them as friends. The three relationships (carers, friends, and family) were incorporated into one dimension because the manner in which participants spoke about these relationships impacting upon their quality of life was fundamentally the same.

### Emotional well-being

A large majority of participants spoke about how their emotional well-being such as general feelings of worry, ‘feeling down’ and anxiety, often associated with everyday experiences affected their quality of life. For some participants, these feelings were linked to the fear of having to leave their own homes and move into residential care. Other participants spoke about experiencing depression as a result of their physical decline which meant they were no longer able to participate in activities or hobbies that they previously enjoyed. Other instances of depression amongst participants were related to family disagreements and fallouts which for some had resulted in complete disconnection from family members which had caused great distress.

### Mobility

Almost all participants spoke about their physical mobility and the limitations they experienced which impacted upon their quality of life. For many participants, these changes meant they had to find ways of adapting to continue to do everyday tasks or activities that they had previously enjoyed. However, some participants experienced major restrictions as a result of their decline in mobility which prevented them entirely from doing what they previously had enjoyed. In general, participants were accepting of changes to their mobility and accepted physical mobility limitations as a normal and inevitable part of the ageing process. However, some participants indicated that they often still felt frustrated by their lack of ability to do what they wanted to do. Many participants discussed the importance of their mobility aids such as scooters, wheelchairs, walking frames and walking sticks to help them get around and carry on doing the things they enjoyed.

### Activities

Involvement in group activities, for example singing groups, craft classes and in independent activities, for example, crosswords, sewing were important to every one of the participants for their quality of life. There were several reasons why they chose to participate. For example, some participants enjoyed the social connections that activities facilitated and their main reason for participating was the social contact they experienced with close friendships often being developed. Other participants spoke about taking part in activities to keep busy as a way of passing time, so the day went quicker. Several participants discussed how doing activities to keep their mind and brain active was particularly important to them as they got older. Role continuity was also raised by some participants with activities linked to previous roles and hobbies helping to maintain their identity. There were also some participants that spoke about their loss in participating in activities, mostly as a result of physical decline, and the upset they experienced from this loss (Table 2).

## Discussion

The QoL dimensions identified in this research are similar to those included in some of the preference-based measures that have been applied within the older population previously [10]. For example, the ICECAP-O has a control dimension similar to the concept in this study identifying levels of independence but uses ‘I am able’ in the item wording reflecting the capability scale it adopts. Our dimension is similar identifying independence through decision-making and control over lives. The ASCOT includes a social connections and participation dimension which aligns with our social connections dimension identifying levels of social contact people experience. Similarly, the EQ-5D covers depression and anxiety, identifying the extent of depression and anxiety individuals experience. Our concept of emotional well-being is similar as it includes happiness and feelings of stress and worry but is described in different terms to the EQ-5D dimension as it does not use the words ‘depression’ or ‘anxiety’ as older people tended to not regularly adopt these during the interview when talking about emotional well-being. Furthermore, feelings of stress and worry amongst older people were often expressed as being related to the fear of moving into residential care which is unique to this population.

Whilst the draft dimensions developed from this research share some similarities with dimensions in existing measures, there are some important differences. For example, the ASCOT consists of a control dimension which is linked to independence but is described in different terms as it identifies levels of control over daily life rather than general feelings of independence and being able to make one’s own decisions. Similarly, the ICECAP-O consists of the dimension attachment which is related to social connections but is different to our dimension as it includes the concept of love in addition to friendships. Whilst the concept of love is no less relevant to people of an older age, it may not reflect the real-life experiences of older people as many are widowed during their later life. This is particularly relevant to older females who on average live longer than males [45]. Our social connections dimension has a broader focus incorporating social relationships with family and friends and connections to the community. The ICECAP-O also includes a security dimension which is partially related to the concept of emotional well-being as it focuses on concerns when thinking about the future. Likewise, the ASCOT includes a dignity dimension identifying if the way in which individuals are treated impacts on how they feel about themselves. However, our concept of emotional well-being is dissimilar to these two dimensions as it covers a generic concept of emotional well-being; specifically identifying feelings of happiness, stress and worry.

Although mobility is included in the EQ-5D, the wording used for the mobility dimension is not age appropriate. Many older people in our study discussed mobility in relation to their ability to get out and about (including with the use of mobility aids if they regularly used them), and these issues are not encapsulated in the way that mobility is described within the EQ-5D instrument as it does not make reference to mobility aids which are often used by older people receiving aged care services. An activities dimension is included in the EQ-5D which focuses on ability to perform usual activities. Likewise, the ICECAP-O has two dimensions that are related to activities, one concentrating on doing things that make you feel valued and the other on enjoyment and pleasure. These activity dimensions are different to our dimension as our dimension focuses more on spending time doing things for enjoyment alone or with other people and therefore has a unique concept of older people’s needs in relation to activities.

Whilst the qualitative approach adopted in this study to identify draft dimensions share some similarities with the development of the ICECAP-O descriptive system there are some important differences. Importantly, the ICECAP-O is a measure of capability and has Sen’s capability approach as its fundamental theoretical foundation. The scoring system for ICECAP-O is anchored upon an absence of capability to full capability scale rather than anchoring on being dead and full health as is usual for generic preference- based measures which generate QALYs [46]. Research investigating the relationship between capability and functioning is in its infancy. However, there are some early indications that whilst these concepts are related, they are separate. Research conducted by Van Leeuwen et al. [47] exploring measurement properties of the EQ-5D-3L, ASCOT and ICECAP-O with older adults found that responses to the EQ-5D-3L were more strongly associated with physical health than were responses to the ICECAP-O and ASCOT instruments.. Conversely, mental health status was more strongly associated with responses to the ICECAP-O, whilst self-perceived QoL and mastery was associated more strongly with responses to the ASCOT. Al-Janabi [48] in his study of 943 family members of meningitis patients, similarly identified that whilst a large proportion of responses indicated that capability equalled functioning (86%) across the dimensions of the ICECAP-A questionnaire, a proportion of responses (12%) demonstrated higher capability than functioning. Participants were more likely to report a difference between their capability and their functioning when their health status was impaired (as indicated by a EQ-5D-5L index score less than 1) as compared to those with unimpaired health status, or if they had caring duties, both groups of people who are likely to be reflected in aged care users. Previous empirical studies have also identified discrepancies between capability and functioning. For example, Bulamu et al. [49] reported relatively high capability in older adults using community aged care services (ICECAP-O mean score 0.76) in comparison with quality of life (EQ-5D mean score 0.47). These identified differences highlight the importance of developing a new measure of quality of life from inception with older people suitable for the aged care context that uses the content and language most often expressed by older people themselves.

Our findings generally concur with those of several previous studies which have demonstrated that the concept of QoL for older people goes beyond health status incorporating broader dimensions of QoL. For example, Ratcliffe et al.’s [16] study comparing the preferences of younger and older people in relation to QoL indicated that older people valued being independent, physically mobile and being in control. Similar research conducted with older adults in a day rehabilitation centre in South Australia found that although older people valued health as important in relation to their QoL, wider dimensions such as independence, control and social relationships were also important [18]. Other research has also highlighted independence and control as key dimensions of QoL amongst older people [13, 17].

### Strengths and limitations

The main strength of this research is the person-centred approach adopted. This method enables the language used by older people to be retained throughout the development of the QoL dimensions which we expect to ultimately result in greater content and face validity, and additionally, making the measure user-friendly. Furthermore, the dimensions have been developed directly from older people receiving community aged care services which means they are of greater relevance to this population. A diverse socio-demographic population broadly representative of older people accessing community aged care services in Australia were recruited across three different Australian states, thus strengthening the validity of the results.

The current study was also designed to be inclusive for older adults living with minor cognitive impairments and/or mild dementia which is a strength of this research as this group are important users of aged care services. Older people living with cognitive impairment and dementia have traditionally been excluded from research of this nature and the development of new preference-based QoL instruments.

The use of one-to-one interviews in a private setting enabled older people to discuss sensitive issues that they may not have felt comfortable discussing in a focus group setting. Most of the language and terminology used by the participants was consistent when describing QoL dimensions but more work will need to be done to establish appropriate wording to ensure the measure is suitable for all older people accessing aged care and to determine the generalisability of the QoL dimensions beyond this context e.g. for older people living independently in the community.

A potential limitation of this study is that the use of cards in the second stage of the interview may have had the potential to influence participant’s responses. The cards were used to initiate further discussion around the dimensions participants had raised in stage one of the interviews. A very small number (*n* = 2) when reading the cards identified a QoL dimension from the cards that was important to them that they had not discussed in the first stage of the interview. However, these data were analysed separately to ensure the dimensions were developed from stage 1 of the interview and therefore any possible influence would be minimal in this respect.

Although a diverse sample of participants was recruited, we recognise that hard to reach older adults may have been excluded from this study. The study does not include respondents who could not communicate in English. Interviewing older adults whose first language was not English was unfortunately outside the scope of this project due to resource limitations. However, it is recognised that, in common with many other developed nations, Australia’s population is culturally and linguistically diverse (CALD). Therefore, future research is planned to identify the extent to which older people from CALD backgrounds value the same QoL dimensions as older people from Anglo/English-speaking populations. It is therefore expected that the QoL measure developed from the current research may be further developed to make it meaningful, acceptable, and suitable to assess the QoL of older people whose first language is not English.

## Conclusion

This study has identified the key QoL dimensions articulated by older people in receipt of community aged care services about what is important to them to experience a good QoL. The five key dimensions identified will be used to inform the development of a new preference-based measure of QoL specific for older people in the aged care context. The dimensions have been developed directly from older people adopting a person-centred approach and therefore have not been influenced by existing literature or other individuals such as carers, family members, or aged care providers. Further work will focus on developing a draft descriptive system to test face validity and psychometric testing to further refine and generate the final descriptive system. The study findings represent a crucial first stage in a multiphase project working in partnership with older people to develop a new preference-based QoL measure for informing quality assessment and economic evaluation in community aged care.

## Data Availability

Not applicable.

## References

[1] Khadka J, Lang C, Ratcliffe J, Corlis M, Wesselingh S, Whitehead C (2019). Trends in the utilisation of aged care services in Australia, 2008–2016 (Report). BMC Geriatrics.

[2] Australian Institute of Health and Welfare. (2019). *Aged care*. Retrieved March 31, 2020, from https://www.aihw.gov.au/reports/australias-welfare/aged-care.

[3] Australian Institute of Health and Welfare. (2018). *Australia's Health. Australia’s health series no. 16. AUS 221*. Retrieved March 20, 2020.

[4] Lancet T (2019). Aged care in Australia falls short. The Lancet.

[5] Bulamu NB, Kaambwa B, Ratcliffe J (2018). Economic evaluations in community aged care: A systematic review. BMC Health Services Research.

[6] Easton T, Milte R, Crotty M, Ratcliffe J (2017). Where's the evidence? A systematic review of economic analyses of residential aged care infrastructure. BMC health services research.

[7] Easton T, Milte R, Crotty M, Ratcliffe J (2016). Advancing aged care: A systematic review of economic evaluations of workforce structures and care processes in a residential care setting. Cost Effectiveness and Resource Allocation.

[8] Makai P, Brouwer WB, Koopmanschap MA, Stolk EA, Nieboer AP (2014). Quality of life instruments for economic evaluations in health and social care for older people: A systematic review. Social Science & Medicine.

[9] Ratcliffe J, Laver K, Couzner L, Cameron ID, Gray L, Crotty M (2010). Not just about costs: The role of health economics in facilitating decision making in aged care. Age and Ageing.

[10] Cleland J, Hutchinson C, Khadka J, Milte R, Ratcliffe J (2019). A Review of the development and application of generic preference-based instruments with the older population. Applied health economics and health policy.

[11] The EuroQol Group (1990). EuroQol: A new facility for the measurement of health-related quality of life. Health Policy.

[12] Herdman M, Gudex C, Lloyd A, Janssen MF, Kind P, Parkin D (2011). Development and preliminary testing of the new five-level version of EQ-5D (EQ-5D-5L). An International Journal of Quality of Life Aspects of Treatment, Care and Rehabilitation - Official Journal of the International Society of Quality of Life Research.

[13] Netten A, Burge P, Malley J, Potoglou D, Towers AM, Brazier J (2012). Outcomes of social care for adults: Developing a preference-weighted measure. Health Technology Assessment.

[14] Coast J, Flynn TN, Natarajan L, Sproston K, Lewis J, Louviere JJ (2008). Valuing the ICECAP capability index for older people. Social Science & Medicine.

[15] van Leeuwen KM, Jansen APD, Muntinga ME, Bosmans JE, Westerman MJ, van Tulder MW (2015). Exploration of the content validity and feasibility of the EQ-5D-3L, ICECAP-O and ASCOT in older adults. BMC Health Services Research.

[16] Ratcliffe J, Lancsar E, Flint T, Kaambwa B, Walker R, Lewin G (2017). Does one size fit all? Assessing the preferences of older and younger people for attributes of quality of life. Quality of Life Research.

[17] Grewal I, Lewis J, Flynn T, Brown J, Bond J, Coast J (2006). Developing attributes for a generic quality of life measure for older people: Preferences or capabilities?. Social Science & Medicine.

[18] Milte CM, Walker R, Luszcz MA, Lancsar E, Kaambwa B, Ratcliffe J (2014). How important is health status in defining quality of life for older people? An exploratory study of the views of older South Australians. Applied Health Economics and Health Policy.

[19] Stevens K (2016). How well do the generic multi-attribute utility instruments incorporate patient and public views into their descriptive systems?. The Patient: Patient-Centered Outcomes Research.

[20] U. S. Department of Health and human services: Food and Drug Administration (2009). Guidance for Industry Patient-Reported Outcome Measures: Use in Medical Product Development to Support Labeling Claims.

[21] Stevens KJ (2010). Working with Children to develop dimensions for a preference-based, generic, pediatric health-related quality-of-life measure. Qualitative Health Research.

[22] Al-Janabi H, Flynn TN, Coast J (2012). Development of a self-report measure of capability wellbeing for adults: the ICECAP-A. Quality of Life Research.

[23] Grewal I, Lewis J, Flynn T, Brown J, Bond J, Coast J (2006). Developing attributes for a generic quality of life measure for older people: preferences or capabilities?. Social Science and Medicine.

[24] Coast J, Flynn TN, Natarajan L, Sproston K, Lewis J, Louviere JJ (2008). Valuing the ICECAP capability index for older people. Social Science and Medicine.

[25] Brod M, Tesler L, Christensen T (2009). Qualitative research and content validity: Developing best practices based on science and experience. An International Journal of Quality of Life Aspects of Treatment, Care and Rehabilitation: Official Journal of the International Society of Quality of Life Research.

[26] McColl E, Fayers P, Hays R (2005). Developing questionnaires. Assessing quality of life in clinical trials.

[27] Stevens K (2011). Assessing the performance of a new generic measure of health-related quality of life for children and refining it for use in health state valuation (Original Research Article). Applied health Economics and Health Policy.

[28] Jorm AF, Mackinnon AJ, Henderson AS, Scott R, Christensen H, Korten AE (1995). The Psychogeriatric Assessment Scales: A multi-dimensional alternative to categorical diagnoses of dementia and depression in the elderly. Psychological Medicine.

[29] Furber C (2010). Framework analysis: A method for analysing qualitative data. African Journal of Midwifery and Women's Health.

[30] Gale NK, Heath G, Cameron E, Rashid S, Redwood S (2013). Using the framework method for the analysis of qualitative data in multi-disciplinary health research. BMC Medical Research Methodology.

[31] Ritchie J, Spencer L, Bryman A, Burgess RG (1994). Qualitative data analysis for applied policy research. Analyzing qualitative data.

[32] Furber CM, Garrod D, Maloney E, Lovell K, McGowan L (2009). A qualitative study of mild to moderate psychological distress during pregnancy. International Journal of Nursing Studies.

[33] Swallow V, Newton J, Van Lottum C (2003). How to manage and display qualitative data using ‘Framework’ and Microsoft ® Excel. Journal of Clinical Nursing.

[34] Connell J, Brazier J, O'Cathain A, Lloyd-Jones M, Paisley S (2012). Quality of life of people with mental health problems: A synthesis of qualitative research. Health Quality of Life Outcomes.

[35] Gorecki C, Nixon J, Madill A, Firth J, Brown JM (2012). What influences the impact of pressure ulcers on health-related quality of life? A qualitative patient-focused exploration of contributory factors. Journal of Tissue Viability.

[36] Hill CL, Baird WO, Walters SJ (2014). Quality of life in children and adolescents with Osteogenesis Imperfecta: A qualitative interview based study (Report). Health and Quality of Life Outcomes.

[37] Markham C, Van Laar D, Gibbard D, Dean T (2009). Children with speech, language and communication needs: Their perceptions of their quality of life. International Journal of Language & Communication Disorders.

[38] Tavernor L, Barron E, Rodgers J, McConachie H (2013). Finding out what matters: Validity of quality of life measurement in young people with ASD. Child: Care, Health and Development.

[39] Stevens K, Palfreyman S (2012). The use of qualitative methods in developing the descriptive systems of preference-based measures of health-related quality of life for use in economic evaluation. Value in Health.

[40] QSR International Pty Ltd. (2018). *NVivo qualitative data analysis software. Version 12*.

[41] IBM Corp. *IBM SPSS Statistics for Windows, Version 25.0*. Armonk, NY: IBM Corp,.

[42] Norman R, Cronin P, Viney R (2013). A pilot discrete choice experiment to explore preferences for EQ-5D-5L health states. Applied Health Economics and Health Policy.

[43] Australian Institute of Health and Welfare. (2019). *Home care packages program data report 1st July–30 September 2019*. Retrieved April 14, 2020, from https://www.health.gov.au/resources/publications/home-care-packages-program-data-reports.

[44] McCaffrey N, Kaambwa B, Currow DC, Ratcliffe J (2016). Health-related quality of life measured using the EQ-5D-5L: South Australian population norms. Health and Quality of Life Outcomes.

[45] Australian Institute of Health and Welfare. (2019). *The Health of Australia's females*. Retrieved April 27, 2020 from https://www.aihw.gov.au/reports/men-women/female-health/contents/who-are.

[46] Brazier J, Ratcliffe J, Salomon J, Tsuchiya A (2017). Measuring and valuing health benefits for economic evaluation.

[47] van Leeuwen KM, Bosmans JE, Jansen APD, Hoogendijk EO, van Tulder MW, van Der Horst HE (2015). Comparing measurement properties of the EQ-5D-3L, ICECAP-O, and ASCOT in frail older adults. Value in Health.

[48] Al-Janabi H (2018). Do capability and functioning differ? A study of U.K. survey responses. Health Economics.

[49] Bulamu N, Kaambwa B, Gill L, Cameron I, Mckechnie S, Fiebig J (2017). Impact of consumer-directed care on quality of life in the community aged care sector. Geriatrics & Gerontology International.

